# Long-term all-cause mortality and cardiovascular outcomes in Scottish children after initiation of renal replacement therapy: a national cohort study

**DOI:** 10.1007/s00467-019-04430-4

**Published:** 2019-12-16

**Authors:** Dinara B. Galiyeva, Caroline A. Jackson, Sarah H. Wild, Susan Burns, David Hughes, Jamie P. Traynor, Wendy Metcalfe, Nynke Halbesma

**Affiliations:** 1 Usher Institute, University of Edinburgh, Edinburgh, UK; 2 Royal Hospital for Children, Glasgow, UK; 3 Scottish Renal Registry, Glasgow, UK

**Keywords:** End-stage renal disease, Dialysis, Transplant, Children, Mortality, Cardiovascular disease

## Abstract

**Background:**

Data on long-term outcomes in children who have received renal replacement therapy (RRT) for end-stage renal disease are limited.

**Methods:**

We studied long-term survival and incidence of fatal and nonfatal cardiovascular disease (CVD) events and determinants of these outcomes in children who initiated RRT between 1961 and 2013 using data from the Scottish Renal Registry (SRR). Linkage to morbidity records was available from 1981.

**Results:**

A total of 477 children of whom 55% were boys, almost 50% had congenital urinary tract disease (CAKUT), 10% received a transplant as the first mode of RRT and almost 60% were over 11 years of age at start of RRT were followed for a median of 17.8 years (interquartile range (IQR) 8.7–26.6 years). Survival was 87.3% (95% confidence interval (CI) 84.0–90.1) at 10 years and 77.6% (95% CI 73.3–81.7) at 20 years. During a median follow-up of 14.96 years (IQR 7.1–22.9), 20.9% of the 381 patients with morbidity data available had an incident of CVD event. Age < 2 years at start of RRT, receiving dialysis rather than a kidney transplant and primary renal disease (PRD) other than CAKUT or glomerulonephritis (GN), were associated with a higher risk of all-cause mortality. Male sex, receiving dialysis rather than a kidney transplant and PRD other than CAKUT or GN, was associated with a higher risk of CVD incidence.

**Conclusions:**

Mortality and CVD incidence among children receiving RRT are high. PRD and RRT modality were associated with increased risk of both all-cause mortality and CVD incidence.

## Introduction

End-stage renal disease (ESRD) in children is rare with median global incidence of renal replacement therapy (RRT) estimated to be nine children per million aged 4–18 years in 2008 [1]. Nevertheless, it is a serious healthcare problem requiring RRT in the form of dialysis or kidney transplantation to sustain life [2]. Recent data from the European Renal Registry (ESPN/ERA-EDTA) show that the incidence of RRT among children aged 0–14 years is around six per million each year [3]. Mortality rates in children receiving RRT are much higher than in the age and sex-matched general population [4]. Cardiovascular disease (CVD) is the leading cause of death in these children, responsible for 23–60% of all deaths [4–7]. Several factors are known to be associated with mortality risk. These include modality at start of RRT, with children who started RRT with dialysis having an almost seven times higher risk of mortality compared to those who received a pre-emptive kidney transplant (HR 6.6, 95% CI 2.9–14.8) [8]. Younger age at start of RRT [4–11], female sex [5] and primary renal disease (PRD) other than congenital anomalies of kidney and urinary tract (CAKUT) [4, 5, 12] have also been found to be associated with increased risk of mortality among children receiving RRT. Studies so far have generally reported 5-year survival [6, 8, 13]. Only limited data exist about longer-term survival [4, 14, 15] and incidence of nonfatal CVD events [9, 11] in individuals who start RRT in childhood. The aims of this study were to describe long-term survival and incidence of fatal and nonfatal CVD events in children after starting RRT and to describe the association between age at initiation of RRT, sex, PRD and RRT modality and these outcomes.

## Materials and methods

### Study population

We included all children (aged <18 years) from the Scottish Renal Registry (SRR) who started RRT between January 1, 1961 and December 31, 2013. The SRR is a national registry that collects individual patient data on date of birth, sex, PRD, start date of RRT, treatment modality at start of RRT, any subsequent changes in treatment modality and vital status for all people starting RRT in Scotland [16].

### Data linkage

**Fig 1:**
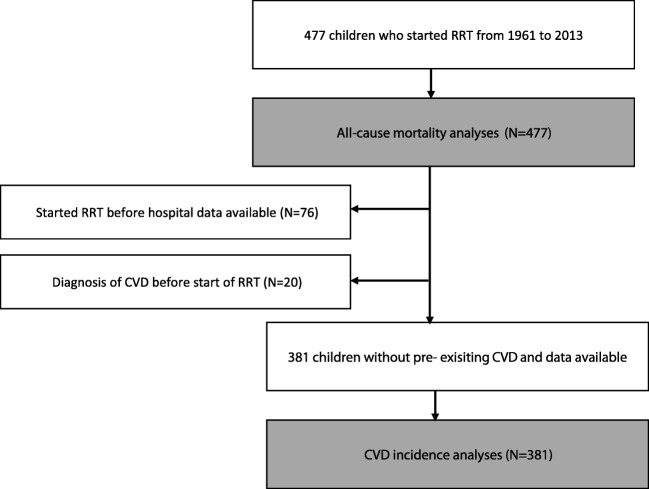
Flow chart describing the study population of children who started renal replacement therapy in Scotland

To describe mortality and CVD incidence, we linked data from National Records of Scotland (NRS) death records and the Scottish Morbidity Records (SMR01) database from Information Services Division (ISD) Scotland with SRR data. The SMR01 database holds data on inpatient and day-case hospital discharges in Scotland from 1981 onwards [17]. Therefore, we selected a sub-cohort of the SRR patients who started RRT from 1981 and 2013 to describe the incidence of fatal and nonfatal CVD events (Fig. 1). We obtained approval for the data linkage and analysis from the Public Benefit and Privacy Panel for Health and Social Care (PBPP) [18] and the SRR steering group. We stored and accessed all datasets according to ISD information governance rules and processes.

### Follow-up period

We followed patients from the start of RRT until the outcome of interest, the end of observation period (December 31, 2015) or loss to follow-up, whichever came first.

### Outcome variables

All-cause mortality and CVD incidence were the outcomes of interest. We defined CVD incidence using the first CVD event after the start of RRT in any position listed in the SMR01 database or in NRS death records. We defined fatal CVD events from the NRS death records from International Classification of Disease (ICD) codes for any circulatory disease. We categorised fatal CVD events as ischaemic heart disease, cerebrovascular disease, heart failure, cardiac arrest/arrhythmias, cardiomyopathy and other diseases of the circulatory system. We did not include codes for other diseases of the circulatory system in the definition of nonfatal CVD events (Table 1).

**Table 1:** ICD-9 and ICD-10 codes used for fatal and nonfatal CVD events

CVD events	Nonfatal CVD events		Fatal CVD events	
	ICD-9	ICD-10	ICD-9	ICD-10
Ischaemic heart disease	410–414	I20–I25	410–414	I20–I25
Cerebrovascular disease	430–438	I60–I69	430–438	I60–I69
Heart failure	428	I50	428	I50
Cardiac arrest/arrhythmias	427	I46–I49	427	I46–I49
Cardiomyopathy	425	I42, I43	425	I42, I43
Other diseases of circulatory system	-	-	390-405, 415-417, 420-424, 426, 429, 440-459	I00–I15, I26–I28, I30–I41, I44, I45, I51, I52, I70–I99

CVD; cardiovascular disease, ICD; international classification of diseases -9 indicates ninth revision used to classify death records and hospital records until 1999, -10 indicates tenth revision used after 1999 to classify death records and hospital records

We categorised patients in accordance with previous publications into the following four age groups at start of RRT: 0–<2 years old, 2–<6 years old, 6–<12 years old and 12–<18 years old [19, 20]. We classified PRD according to the ERA-EDTA coding system [21] and grouped it as CAKUT, glomerulonephritis (GN) and other. The other category includes cystic kidney disease, hereditary nephropathy, ischaemic renal failure, haemolytic-uraemic syndrome (HUS), metabolic disorders, vasculitis and miscellaneous. We categorised initial type of RRT as haemodialysis (HD), peritoneal dialysis (PD) and pre-emptively transplanted (pre-Tx). We classified RRT patterns during follow-up into four categories: started on HD and not transplanted during follow-up (HD + Tx-), started on PD and not transplanted during follow-up (PD + Tx-), pre-Tx and transplanted ever after starting RRT on dialysis (D + Tx+).

### Statistical methods

We calculated crude all-cause mortality and CVD incidence rates per 100 person years of follow-up. We used unadjusted cumulative incidence competing risk (CICR) analysis to estimate the risk of mortality and cardiovascular disease by initial RRT modality [22] and accompanying 95% confidence intervals [23]. Cause-specific Cox proportional hazard regression analyses were used to describe survival probabilities and risk of outcomes by age at start of RRT, sex, PRD and RRT modality. Patients were censored after experiencing the outcome of interest as only first occurring events are taken into account. We evaluated the proportional hazard assumption graphically using log minus log plots. We adjusted the estimates of associations from Cox proportional hazard models for possible confounding factors including age at start of RRT, sex, PRD, initial RRT modality and period of start of RRT (1961–1990, 1991–2000 and 2001–2013). Data were complete for all variables included in the Cox regression analyses.

### Sensitivity analysis

Since children who receive pre-emptive transplants (i.e. those who do not receive dialysis prior to transplant) generally have better survival than children who initially receive dialysis, we performed a sensitivity analysis excluding children who received a pre-emptive transplant from the Cox regression analysis. We compared the results of this sensitivity analysis with the results of the main analyses.

## Results

### Patient characteristics

Table 2 presents the demographic characteristics of the 477 children included in the all-cause mortality analyses and the 381 children included in the analyses of CVD incidence. The characteristics of both cohorts show that the proportion of boys was higher than girls and the majority of the patients started RRT when they were older than 12 years of age. Furthermore, CAKUT was the most common underlying PRD, and almost 90% of patients started RRT on dialysis, and only 10% of children received a pre-emptive transplant.

**Table 2:** Characteristics of patients included in the analyses

	All-cause mortality cohort	CVD incidence cohort
Characteristics	N (%)	N (%)
N included	477	381
Sex		
Male	264 (55.3)	218 (57.2)
Age at start of RRT (years)		
0–<2	30 (6.3)	29 (7.6)
2–<6	50 (10.5)	47 (12.3)
6–<12	113 (23.7)	90 (23.6)
12–<18	284 (59.5)	215 (56.4)
PRD		
CAKUT	229 (48.0)	180 (48.6)
GN	80 (16.8)	59 (15.5)
Other	168 (35.2)	137 (36.0)
Initial RRT modality		
HD	220 (46.1)	155 (40.7)
PD	207 (43.4)	180 (47.2)
Tx	50 (10.5)	46 (12.1)
RRT modality during follow-up		
D + Tx+	371 (77.8)	289 (75.9)
Pre-Tx	50 (10.5)	46 (12.1)
HD + Tx-	30 (6.3)	24 (6.3)
PD + Tx-	26 (5.5)	22 (5.8)

*CVD* cardiovascular disease, *RRT* renal replacement therapy, *PRD* primary renal disease, *CAKUT* congenital anomalies of kidney and urinary tract, *GN* glomerulonephritis, *HD* haemodialysis, *PD* peritoneal dialysis, *Tx* transplanted. *PD + Tx* started on PD and not transplanted during follow-up, *HD + Tx* started on HD and not transplanted during follow-up, *pre-Tx* pre-emptive transplant, *D + Tx +* started on dialysis (PD or HD) and received a transplant during follow-up

### Survival after the start of renal replacement therapy

**Fig 2:**
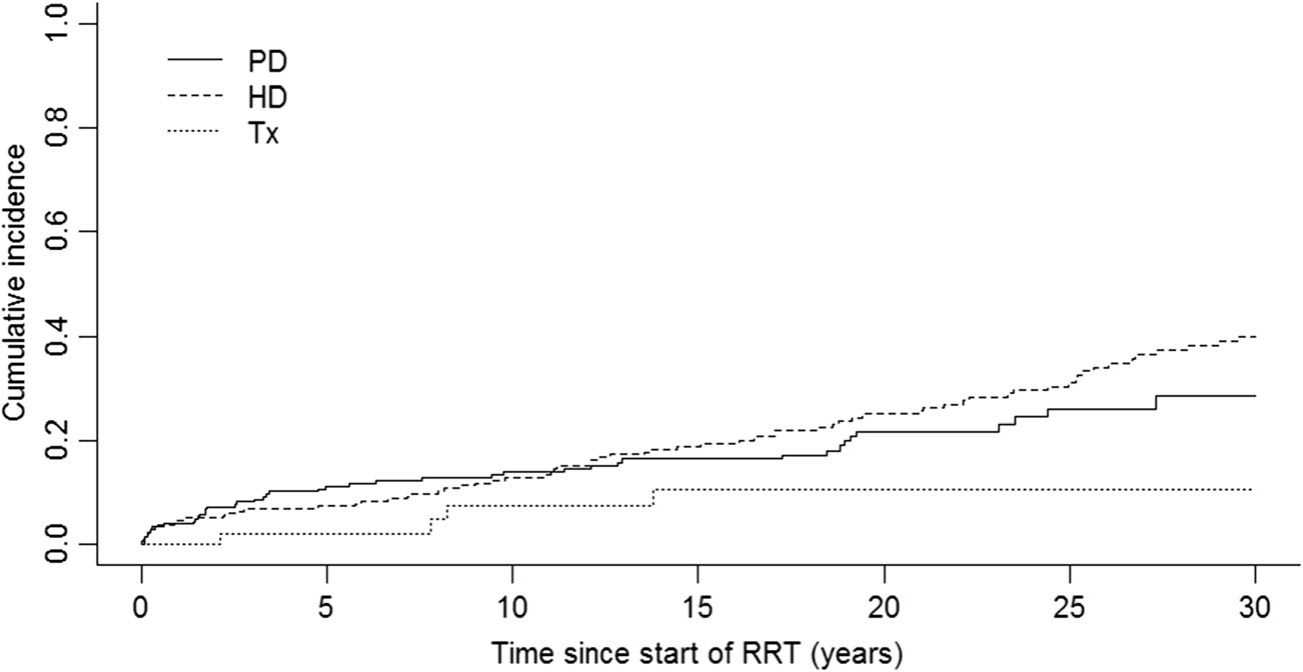
Cumulative incidence curves for mortality in Scottish children after start of renal replacement therapy between 1961 and 2013 by initial renal replacement therapy modality

In total, 477 children were followed for a median of 17.8 years (IQR 8.7–26.6 years), giving a total follow-up time of 8710 person years. Median follow-up time was longest for patients starting RRT on HD (21.5 year), followed by PD (17.2 years), and pre-emptive transplant patients had the shortest median follow-up time of 14.2 years. During the total study period, 125 patients died resulting in a crude all-cause mortality rate of 1.44 (95% CI 1.19–1.70) per 100 person years. Of these deaths, 36.5% were due to a known cardiovascular cause, 11.2% of the deaths were due to infections, 5.6% due to malignancies, and 2.4% caused by haemorrhages*.* After 10 years of follow-up, the overall survival after the start of RRT was 87.3% (95% CI 84.0–90.1), and 337 were still alive with follow-up available, after 20 years overall survival was 77.6% (95% CI 73.3–81.7), and 209 children were still followed-up. Mortality was lower in children who received a pre-emptive transplant compared to children who received HD as their first RRT (0.55, 95% CI 0.01–1.09 per 100 person years and 1.68, 95% CI 1.31–2.06 per 100 person years, respectively) or children who started with PD (1.29, 95% CI 0.91–1.67 per 100 person years) although a statistically significant difference was only observed for the comparison between children receiving a pre-emptive transplant and those receiving HD as their first RRT. Figure 2 presents cumulative incidence curves based on unadjusted CICR analyses. This figure shows that after 10 and 20 years, follow-up survival among patients who started on HD was 87.1% (95% CI 82.2–91.3) and 75.0% (95% CI 68.6–81.0); on PD these figures were 86.1% (95% CI 80.9–90.5) and 78.3% (95% CI 71.6–84.4). Patients who received a pre-emptive transplant showed higher survival (after 10 years 92.5% (95% CI 81.4–98.1) and 20 years 89.4% (95% CI 76.9–96.8)).

### Cardiovascular disease incidence after the start of renal replacement therapy

The cohort included in the CVD incidence analyses of 381 children was followed for a median of 14.96 (IQR 7.05–22.87) years with a total follow-up of 5739 person years. The mean age at the first CVD event was 22.1 (standard deviation ± 9.4) years. In total 80 (20.9%) children experienced a CVD event of which 20 had a fatal event. The most common types of CVD were cerebrovascular disease (N = 29) followed by heart failure (N = 13), cardiac arrest/arrhythmias (N = 13) and other (N = 12 of which 55% were due to cardiomegaly and 27% were classified as endocarditis), ischaemic heart disease (N = 11) and cardiomyopathy (N = 2).

**Fig 3:**
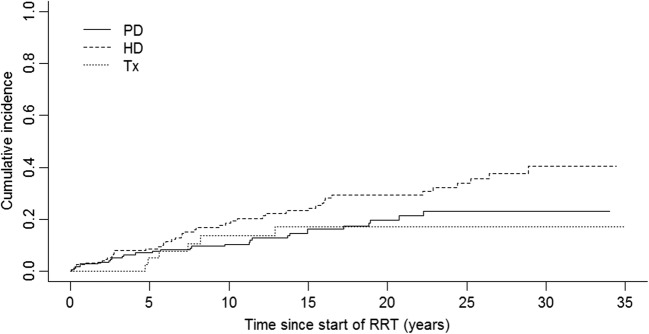
Cumulative incidence curves for cardiovascular disease in Scottish children after start of renal replacement therapy between 1981 and 2013 by initial renal replacement therapy modality

The overall CVD incidence was 1.39 (95% CI 1.09–1.70) per 100 person years. CVD incidence was lowest among pre-Tx children (0.93, 95% CI 0.19–1.67 per 100 person years) compared with children who started with HD (1.87, 95% CI 1.33–2.43) per 100 person years) or PD (1.08, 95% CI 0.68–1.47 per 100 person years) (Fig. 3), but these differences were not statistically significant. Figure 3 shows the cumulative incidence curves based on unadjusted CICR analyses.

### Associations between determinants and all-cause mortality and cardiovascular disease incidence

Table 3 presents the results of the cause-specific Cox proportional hazard analyses showing associations between age at start of RRT, sex, PRD and RRT modality with all-cause mortality and CVD incidence. Starting RRT at a very young age (0–2 years), PRD other than CAKUT or GN and receiving dialysis rather than a kidney transplantation as the initial RRT modality are significantly associated with increased risk of all-cause mortality. Furthermore, these results show that patients who never received a transplant have an increased mortality risk compared with patients who are pre-emptively transplanted or who received a transplant during follow-up. Sex was not associated with all-cause mortality, but boys/men had an increased risk of CVD compared to girls/women. Other factors associated with increased risk of CVD were PRD other than CAKUT or GN and receiving no transplant during follow-up. Similar results were obtained in a sensitivity analysis excluding 50 children who received a pre-emptive transplant .

**Table 3:** Crude and adjusted hazard ratios for associations between determinants and all-cause mortality and cardiovascular disease incidence

Variable	All-cause mortality		CVD incidence	
	Crude HR (95% CI)	Adjusted HR (95% CI)	Crude HR (95% CI)	Adjusted HR (95% CI)
Age at start of RRT^a^				
0–<2	2.41 (1.27–4.57)	2.87 (1.42–5.82)	0.73 (0.27–2.02)	0.89 (0.31–2.59)
2–<6	0.93 (0.60–1.44)	1.39 (0.67–2.67)	0.54 (0.23–1.25)	0.77 (0.32–1.91)
6–<12	0.97 (0.50–1.89)	1.04 (0.66–1.64)	0.67 (0.38–1.17)	0.79 (0.44–1.41)
12–18	1.00	1.00	1.00	1.00
Sex^b^				
Males	1.27 (0.89–1.82)	1.31 (0.92–1.88)	1.51 (0.96–2.38)	1.76 (1.11–2.79)
Females	1.00	1.00	1.00	1.00
PRD^c^				
GN	0.99 (0.61–1.65)	1.01 (0.61–1.69)	1.17 (0.63–2.18)	1.12 (0.59–2.19)
Other	1.45 (0.98–2.13)	1.50 (1.01–2.22)	1.46 (0.90–2.37)	1.67 (1.01–2.75)
CAKUT	1.00	1.00	1.00	1.00
Initial RRT modality^d^				
HD	2.97 (1.08–8.07)	2.57 (0.92–7.18)	2.04 (0.86–4.78)	1.73 (0.73–4.11)
PD	2.41 (0.87–6.70)	2.06 (0.73–5.85)	1.17 (0.49–2.82)	1.38 (0.56–3.41)
Pre-Tx	1.00	1.00	1.00	1.00
RRT modality during follow-up^e^				
HD + Tx-	14.4 (8.01–25.77)	20.03 (10.77–37.25)	5.51 (2.14–14.16)	6.20 (2.29–16.80)
PD + Tx-	17.3 (9.71–30.76)	20.69 (10.73–39.89)	5.50 (2.41–12.55)	5.81 (2.50–13.50)
Pre-Tx	0.55 (0.20–1.51)	0.68 (0.25–1.86)	0.74 (0.32–1.71)	0.69 (0.30–1.61)
D + Tx+	1.00	1.00	1.00	1.00

*CVD* cardiovascular disease; *HR* hazard ratio, *CI* confidence interval; *RRT* renal replacement therapy; *PRD* primary renal disease; *GN* glomerulonephritis, *CAKUT* congenital anomalies of kidney and urinary tract; *HD* haemodialysis; *PD* peritoneal dialysis; *pre-Tx* pre-emptively transplanted; *HD + Tx* started on HD and not transplanted during follow-up, *PD + Tx* started on PD and not transplanted during follow-up, *D + Tx +* transplanted ever after starting on dialysis. Only patients with complete data included in unadjusted and adjusted analyses; adjusted for ^a^ sex, PRD, type of RRT at start and period of start of RRT; ^b^ age at start of RRT, PRD, type of RRT at start and period of start of RRT; ^c^age at start of RRT, sex, type of RRT at start and period of start of RRT; ^d^ age at start of RRT, sex, PRD and period of start of RRT; ^e^ age at start of RRT, sex, PRD and period of start of RRT

## Discussion

### Summary of principal findings

In this study, we described survival and CVD incidence in children who started RRT after 1961 in Scotland and investigated how key patient and treatment factors were associated with these outcomes. Of the 477 children who started RRT in Scotland between 1961 and 2013, 125 died during a total of 8.710 person years of follow-up. Unadjusted cumulative survival for the cohort was 87.3% at 10 years and 77.6% at 20 years. Survival was highest in patients who received a pre-emptive transplant and lowest in patients who started RRT on HD and did not receive a transplant during follow-up. Other factors associated with poorer survival were underlying PRD other than CAKUT or GN and starting RRT at a very young age. During a median follow-up of almost 15 years, 21% of the patients developed incident CVD with the most common type being cerebrovascular disease. CVD incidence was not significantly associated with initial mode of RRT. Boys/men had an increased risk of CVD incidence compared to girls/women. PRD other than CAKUT or GN and receiving dialysis rather than a kidney transplantation were associated with increased risk of CVD.

### Comparison of all-cause mortality with previous studies

Our findings were similar to the 86% cumulative 10-year survival reported from a Canadian cohort including 843 children starting RRT between 1992 and 2007 [15]. However, McDonald and Craig reported slightly lower survival figures based on older data from the Australian and New Zealand (ANZDATA) registries collected between 1963 and 2002 [4], and the ERA/EDTA has published higher overall long-term cumulative survival figures based on recent data from Europe [24]. The variation of these survival rates can at least partly be explained by differences in study period included in the analyses. For example, data from ANZDATA showed increased survival [4] due to availability of effective treatments [24].

Several other reports have studied the association between key patient and treatment factors and all-cause mortality. Consistent with the findings of previous studies, we found that the youngest age group (<2 years at start of RRT) had the highest mortality [5, 15, 24].

Unsurprisingly, we found the highest survival among children who received a pre-emptive transplants and lowest survival among patients who never received a transplant [25]. This finding is in line with earlier studies [26]. Although this finding supports the likely benefit of transplant, there is also the strong possibility that failure to receive a transplant may also be a marker of high risk of mortality due to coexistent conditions that preclude listing for transplantation.

We found the highest all-cause mortality in children with an underlying PRD other than CAKUT or GN which is consistent with reports from a cohort including Canadian infants [15] and reports based on USRDS data [5].

We found that boys had an increased risk of all-cause mortality compared with girls; however this effect was not statistically significant. In contrast a study using USRDS data including both dialysis and transplant patients showed a higher mortality among girls after a median follow-up of 7 years (HR 1.36 (95% CI 1.25–1.50)). Sex disparities in access to (pre-emptive) transplantation, health insurance and underlying PRD only partly explained the difference in survival [27]. A few other studies have shown higher mortality among girls [5, 28], but a short follow-up or only including dialysis patients makes comparison with our results difficult.

### Comparison of cardiovascular outcomes with previous studies

It is well-known that similar to results from the adult RRT population, the proportion of deaths in the paediatric RRT population attributed to CVD is high [7, 8, 14, 27, 29]. However, there are only limited data available on CV outcomes including CV morbidity. A few studies have reported on fatal and nonfatal CVD, but these studies were limited to a short follow-up, a selected population and lacking detailed specification of the type of CV outcomes [9, 29].

One of the few sources reporting the incidence of CVD events is the USRDS. Data from this source showed that first-year CVD hospitalisation are common (rates of 63 per 1000 patient years from 2005 to 2009 and 42 from 2010 to 2014). The highest risk of CVD hospitalisation occurred among children on dialysis which is in agreement with our results. Chavers et al. reported that around a third of the patients who started dialysis between 1990 and 1997 developed a cardiac-related event. Of these events, the majority were arrhythmias, valvular heart disease, cardiomyopathy and cardiac arrests. Children in the oldest age group (15–19 years), blacks and girls, had the highest rates of cardiac-related events [11]. Different inclusion criteria and follow-up make it difficult to directly compare these findings with our results. A possible explanation for the overall lower incidence of CVD events we found (21% compared with 30% based on USRDS data) might be the fact that our population is predominantly Caucasian. Previous studies have reported a high prevalence of a variety of intermediate CV outcomes and CV abnormalities such as left ventricular hypertrophy, coronary calcifications, arrhythmias and significantly increased carotid intima media thickness in children and young adults who started RRT in childhood [30–35]. These results are in agreement with our findings of a high incidence of CVD after long-term follow-up during young adult age (mean age of first CVD event of 21 years) and increased CVD risk among both dialysis and transplant patients compared with general population.

Furthermore, high prevalence of traditional CV risk factors and newer risk factors such as C-reactive protein among dialysis and transplanted patients is reported [27]. For example, a cross-sectional analysis of prevalent RRT patients in the United Kingdom has shown that 75% of patients younger than 18 years old have one or more CV risk factor (obesity, hypertension, hypercholesterolaemia), and one in ten have all three risk factors [36]. These findings raise the expectation of high incidence of fatal and nonfatal CVD incidence in this population.

### Strengths and limitations of the study

In our study, data from all incident paediatric RRT patients in Scotland are included. A novel data linkage with death and hospital admission data offered the opportunity to describe fatal and nonfatal incident CVD events as well as all-cause mortality during follow-up period into adulthood. The study cohort is extremely heterogeneous because all patients who started RRT in Scotland in a certain period are included. This has resulted in a cohort which covers several decades and includes both dialysis and transplant patients (pre-emptively and transplanted during follow-up), a variety of PRDs, infants, children and adolescents resulting in a diverse case mix. We were therefore able to investigate the effect of these diverse characteristics on mortality and CVD incidence and found that the sensitivity analysis excluding children who received a pre-emptive transplant (N = 50) found similar results to the main analysis.

A key limitation is the relatively small sample size despite this study being a population wide study including all incident RRT patients in Scotland over a 50-year calendar period. The small sample size has resulted in low statistical power and precision and limited ability to perform potentially interesting analyses in subgroups of the cohort. Future studies including a larger cohort will offer opportunities to look at analyses stratified by underlying PRD and the impact of time (different types) of RRT on cardiovascular risk in children after initiating RRT. Although important possible confounding variables are taken into account, information on existing comorbidities is not available.

A final potential limitation is that data on causes of death and hospital admissions before 1981 are not available, and therefore it might be possible that a small proportion of the children currently classified as having new onset CVD had an earlier CVD event prior to 1981.

## Conclusion and clinical implications

In summary, our study shows that 87% and 78% of children who started RRT in Scotland between 1961 and 2013 were still alive after 10 and 20 years of follow-up. Long-term survival was highest in patients who receive a pre-emptive transplant, who have CAKUT or GN as PRD and who start RRT at older age. After a median follow-up of 13 years, a fifth of the children developed incident CVD. Male sex, dialysis as treatment modality and PRD other than CAKUT or GN were associated with higher risk of CVD. It is important that physicians providing care to young adults who started RRT during childhood are aware of the implications of childhood illness upon cardiovascular risk. Furthermore, our results showed that patients who received a pre-emptive transplant had a lower risk of all-cause mortality and CVD outcomes. Recent data from the UK Renal Registry showed that less than a quarter of paediatric RRT patients received a pre-emptive transplant [33]. To prevent early mortality and CVD pre-emptive transplantation should be promoted.

Our results strongly suggest that the effectiveness of monitoring and treating CV risk factors children on RRT and young adults who started RRT during childhood should be assessed. The results of the Dutch LERIC cohort have shown promising results of intensified antihypertensive and antidyslipidaemic therapy in young adults who started RRT in childhood. In this cohort of 249 patients who started RRT in childhood and survived into adulthood (follow-up of 25.5 years), a clear decrease in CV mortality along with strict CV management and reversal of CV risk factors was observed [30, 31]. A review published in 2017 describes the under-recognised and under-treated burden of hypertension in transplant patients [37]. In addition another study has reported on contradicting guidelines and differences in policies and treatment of hypertension in Dutch paediatric transplant patients [38]. This evidence together with our reported increased and ongoing CVD risk in paediatric patients underlines the importance of further research to provide evidence to develop specific guidelines for optimal treatment of paediatric RRT patients.

## Data Availability

The datasets generated and analysed during the current study are not publicly available due to the identifiable nature of the data. All data is stored and analysed in a ‘Safe Haven’ environment.
